# Modulation of postprandial lipaemia by a single meal containing a commonly consumed interesterified palmitic acid-rich fat blend compared to a non-interesterified equivalent

**DOI:** 10.1007/s00394-016-1284-z

**Published:** 2016-08-10

**Authors:** Wendy L. Hall, Sara Iqbal, Helen Li, Robert Gray, Sarah E. E. Berry

**Affiliations:** Diabetes and Nutritional Sciences Division, King’s College London, Franklin-Wilkins Building, 150 Stamford Street, London, SE1 9NH UK

**Keywords:** Interesterified fat, Palm stearin, Palm kernel, Postprandial lipaemia

## Abstract

**Purpose:**

Interesterification of palm stearin and palm kernal (PSt/PK) is widely used by the food industry to create fats with desirable functional characteristics for applications in spreads and bakery products, negating the need for *trans* fatty acids. Previous studies have reported reduced postprandial lipaemia, an independent risk factor for CVD, following interesterified (IE) palmitic and stearic acid-rich fats that are not currently widely used by the food industry. The current study investigates the effect of the most commonly consumed PSt/PK IE blend on postprandial lipaemia.

**Methods:**

A randomised, controlled, crossover (1 week washout) double-blind design study (*n* = 12 healthy males, 18–45 years), compared the postprandial (0–4 h) effects of meals containing 50 g fat [PSt/PK (80:20); IE vs. non-IE] on changes in plasma triacylglycerol (TAG), glucose, glucose-dependent insulinotropic polypeptide (GIP), peptide YY (PYY), insulin, gastric emptying (paracetamol concentrations) and satiety (visual analogue scales).

**Results:**

The postprandial increase in plasma TAG was higher following the IE PSt/PK versus the non-IE PSt/PK, with a 51 % greater incremental area under the curve [mean difference with 95 % CI 41 (23, 58) mmol/L min *P* = 0.001]. The pattern of lipaemia was different between meals; at 4-h plasma TAG concentrations declined following the IE fat but continued to rise following the non-IE fat. Insulin, glucose, paracetamol, PYY and GIP concentrations increased significantly after the test meals (time effect; *P* < 0.001 for all), but did not differ between test meals. Feelings of fullness were higher following the non-IE PSt/PK meal (diet effect; *P* = 0.034). No other significant differences were noted.

**Conclusions:**

Interesterification of PSt/PK increases early phase postprandial lipaemia (0–4 h); however, further investigation during the late postprandial phase (4–8 h) is warranted to determine the rate of return to baseline values.

**Trial registration number:**

Clinicaltrials.gov as NCT02365987.

**Electronic supplementary material:**

The online version of this article (doi:10.1007/s00394-016-1284-z) contains supplementary material, which is available to authorized users.

## Introduction

The process of random interesterification is extensively used by the food industry to create fats with desirable functional characteristics for use in spreads and bakery products. This process avoids the use of *trans* fatty acids and results in a fat with a lower saturated fatty acid (SFA) content than traditional hard fats. Interesterification of fats, which may be carried out by random and directed, chemical or enzymatic processes, rearranges fatty acids on their glycerol moiety within the triacylglycerols (TAG) species, thereby changing their TAG structure [stereospecific (*sn*)-positional composition] and their melting properties [1]. Randomly interesterified (IE) fat blends rich in palmitic acid (used in the European market) and stearic acid (used in the North American market) are most commonly used by food manufacturers, and current intakes are estimated to be 2–10 % of daily energy intake. However, despite their widespread use, there has been little research on the health effects of the most commonly consumed palmitic acid-rich IE fats.

The association between dietary fatty acids and cardiovascular disease (CVD) risk has been extensively studied, and predictive equations on their blood lipid effects have been validated [2]. However, whilst the total fatty acid composition of IE fats is identical to their non-IE fat, the *sn*-positional composition and physical characteristics are different, making direct comparisons to non-IE fats invalid. Indeed, it is believed that differences in *sn*-positional composition between some animal and plant fats may explain their divergent effects on atherogenicity despite similar fatty acid compositions. SFA is mainly present in the *sn*-1 and *sn*-3 positions of TAG molecules in vegetable oils, but in animal fats, they are predominantly in the *sn*-2 position. Random interesterification of vegetable oils therefore increases the proportion of SFA in the *sn*-2 position. Animal studies have demonstrated that consumption of TAG-containing palmitic acid in the *sn*-2 position promotes atherogenesis to a greater extent than TAG-containing palmitic acid in the *sn*-1 or *sn*-3 positions [3–5]. However, human dietary intervention studies have not demonstrated that there are any chronic effects of *sn*-positional composition or interesterification of palmitic acid-rich plant fats (whereby the proportion of palmitic acid in the *sn*-2 position is increased) on blood lipids or insulin sensitivity [6–10]. Previous research from our group and others on the acute effects of IE palmitic acid-rich fats (palm oil/olein) consistently shows that IE fats reduce postprandial lipaemia [11–14], in addition to a concurrent reduction in incretin gut hormone responses [15] but do not influence postprandial glucose and insulin concentrations, when compared to the non-IE fat equivalent or a control oil. However, these previously studied fats have little commercial or health relevance, as they are rarely used as an ingredient by the food industry because they do not possess suitable functional properties. The most commonly used IE fats in commercially available foods are blends of plant oils/plant oil fractions, whereas single fat sources, such as palm oil or palm olein oils, are rarely included in industrially processed foods. Blends of palmitic acid- and lauric acid-rich IE fats are the most commonly used IE fats in the European food industry. These fats are generally derived from palm fruits and include palm oil fractions such as palm stearin (PSt) (high in palmitic acid) and, in smaller proportions, palm kernel oil (PK) or coconut oil (both rich in lauric acid). The most commonly consumed IE fat in Europe consists of a blend of IE PSt/PK (typically 80:20) to create a ‘hardstock’ which is blended with vegetable oils (rapeseed or linseed) in varying amounts to create the desired functionality, depending upon the application.

It is now well known that an elevated non-fasting TAG (postprandial) concentration is an important risk factor for both CVD and other chronic metabolic conditions such as fatty liver, insulin resistance and type 2 diabetes. Three large prospective cohort studies have established non-fasting TAG as a significant predictor of CVD risk [16–18]. The mechanisms linking postprandial lipaemia to risk of atherosclerotic CVD underpin many of the observed chronic effects of dietary fats on CVD risk, including effects on lipoprotein remodelling, oxidative stress, inflammation and endothelial dysfunction [19]. Although we spend most of our day in the postprandial state (~18 h), most studies investigating the effects of dietary fat on CVD risk markers have focused on fasting lipid profiles. Therefore, determining an individual’s postprandial response to different dietary fats is an important tool for predicting cardio-metabolic risk and understanding their chronic effects.

The primary aim of the current study was therefore to investigate the effect of one of the most commonly consumed IE fat blends on postprandial lipaemia. To gain a better understanding of why IE fats elicit differences in lipaemia, a secondary aim was to investigate the effects on incretin gut hormones, satiety, gastric emptying, glucose and insulin concentrations. It is hypothesised that IE PSt/PK will reduce the postprandial lipaemic response compared to non-IE PSt/K and that this may be related to a delay in gastric emptying with subsequent effects on satiety, glycaemia and incretin hormones.

## Materials and methods

### Participants

Ethical approval for the study was obtained from the Research Ethics Committee of King’s College London (CREC BDM/14/15-26), and written informed consent was provided by participants. The study was conducted in accordance with the ethical standards laid down in the 1964 Declaration of Helsinki and its later amendments. The trial was registered at clinicaltrials.gov as NCT02365987. Healthy adult males (aged 18–45 years) were recruited via advertisements and circular emails within King’s College London which commenced in February 2015 and completed in April 2015. The participants attended a screening visit at the Metabolic Research Unit (MRU) at the Diabetes and Nutritional Sciences Division, King’s College London following an overnight fast. Their weight, height, waist circumference, percentage body fat, seated blood pressure, haematology, glucose, insulin, liver function and lipid profile were measured. Participants were requested to complete a 3-day diet diary to assess their habitual nutritional intakes using Nutritics Ltd, Ireland. Exclusion criteria were as follows: medical history of myocardial infarction, angina, thrombosis, stroke, cancer, liver or bowel disease or diabetes, body mass index <18 or >35 kg/m^2^, plasma cholesterol ≥7.5 mmol/L, plasma TAG >3 mmol/L, plasma glucose >7 mmol/L, blood pressure ≥160/100 mmHg, current use of antihypertensive or lipid lowering medications, alcohol intake exceeding a moderate intake (>28 units/week) and current cigarette smoker. Sample size calculations were based on 10 participants completing at 90 % power, *P* < 0.05 to detect a 0.54 mmol/L difference in peak plasma TAG concentrations with a SD of differences of 0.5 mmol/L using data from Sanders et al. [12].

### Study design

A randomised, double-blind, crossover design was used to compare two test meals, with a minimum 1-week washout period. Each test meal consisted of a muffin, custard (Birds Eye, Premier Foods, UK) and a milkshake and provided 3.5 MJ (832 kcal), 15 g protein (7 % E), 81 g carbohydrate (37 % E), 52 g (50 g from test fat) fat (56 % E) and 1.5 g non-effervescent paracetamol. The paracetamol was added to measure rates of gastric emptying [20]. The muffin was cut into small bite-sized chunks and evenly covered with the custard mix into which the paracetamol had been dissolved. The test fats were: non-IE PSt/PK and IE PSt/PK blended at a ratio of 80:20 PSt/PK, made from the same batch of oil (Archer Daniels Mills, Erith, UK). Both fat blends had a similar fatty acid composition, but the proportions of palmitic acid at the *sn*-2 position were 36 mol% in non-IE PSt/PK and 55 mol% in IE PSt/PK (Table 1). Measurement of the solid fat content by NMR [European laboratories of Archer Daniel Mills (Hamburg Germany)] of non-IE PSt/PK and IE PSt/PK gave values of 55 and 62 % at 20 °C, 33 and 34 % at 30 °C, 24 and 21 % at 35 °C and 17 and 11 % at 40 °C, respectively.

**Table 1:** Fatty acid composition of the experimental fats (total) and the proportions of fatty acids in the *sn*-2 position of the dietary triacylglyerols (mol%)

Fatty acid	Non-IE PSt/PK	IE PSt/PK
12:0		
Total	6.8	6.5
*sn*-2	12.7	12.7
14:0		
Total	4.0	4.4
*sn*-2	4.9	5.3
16:0		
Total	52.1	51.9
*sn*-2	36.0	54.7
18:0		
Total	4.7	4.9
*sn*-2	1.4	3.7
18:1n-9		
Total	26.5	26.3
*sn*-2	36.9	19.6
18:2n-6		
Total	5.1	5.2
*sn*-2	8.2	4.0

The allocation of treatment was blinded from both the investigators and the study participants. On the day preceding each test meal, participants were told not to participate in strenuous exercise and to avoid alcohol, caffeine and foods high in fat. They were provided with a standardised low fat meal (2–3 MJ containing <10 g fat) to consume as their evening meal, which they were required to consume before 22:00 h and then to avoid eating or drinking anything, except water. Participants attended the MRU between 08:00 and 10:00 h the following morning. A cannula was inserted into the forearm (antecubital vein), and the test meal was consumed within 10 min. Further venous blood samples were collected regularly until 4 h following the test meal. Participants had access to water to sip as required over the 4 h. Visual analogue scales (VAS) were completed by participants at fasting and 30-min intervals postprandially to measure appetite; ‘how hungry do you feel?’; ‘how strong is your desire to eat?’; and ‘how full do you feel?’. Following the 4 h blood sample, they were given an ad libitum lunch meal which was selected from their second preference from a choice of three meals. Each meal contained 3000 kcal (1.25 mJ) and matching macronutrient profiles (48 % carbohydrate, 19 % protein and 28 % fat) and energy density (approximately 1.6 kcal/g). Participants ate until they were full, and the meals were weighed before and after eating.

### Analytical methods

Test oils were analysed for their fatty acid composition and the *sn*-2 fatty acid composition. The fatty acid composition of the oil and the test oil TAG-derived 2-mono-acylglycerol (2-MAG) were analysed following HCl-catalysed methylation to form methyl esters by capillary gas liquid chromatography (GLC) using a BP75 column (25 m × 220 µm × 0.25 µm; SGE Analytical Science, VIC, Australia) on an Agilent 6890 (Agilent Technologies, Cheshire, UK). Fatty acid methyl standards were obtained from Sigma (Poole, Dorset). The proportions of fatty acids in the *sn*-2 position of the TAG were determined following incubation of the TAG with porcine lipase (Type VI-S, from porcine pancreas, EC 3.1.1.3, 100,000 units, Sigma, Poole, Dorset, UK), separation of the 2-MAG and analysis of its fatty acid composition [13].

Blood samples for TAG and insulin were collected into serum separator tubes and allowed to stand at room temperature for 15 min, then centrifuged, and serum collected and stored frozen at −40 °C until analysis. Samples for plasma glucose concentrations were collected into fluoride oxalate vacutainers and kept on ice for a maximum of 15 min, before being centrifuged. Enzymatic assays were used to determine concentrations of TAG and glucose concentrations (ILAB, Instrumentation Laboratories, Warrington, Cheshire, UK) using the GPO-PAP assay and GOD-PAP assay, respectively (Roche Diagnostics Limited, UK), on an ILAB-650 analyzer. Samples for glucose-dependent insulinotropic polypeptide (GIP) and peptide YY (PYY) analysis were collected into 4 mL EDTA vacutainers containing 100 μL of trasylol (10,000 KIU/mL, Nordic Pharma Ltd, Reading, UK) and kept on ice up to 15 min, followed by centrifugation and plasma storage at −80 °C until analysed using a sandwich ELISA and a radioimmunoassay kit (Linco Research, MO, USA), respectively, at Kings College Hospital (KCH). Insulin concentrations were analysed on the Immulite 2000 analyser using the Siemans Advia Centaur two-site sandwich immunoassay (Siemans Medical Solution Diagnostics Europe Ltd, UK) at KCH. Paracetamol concentrations were analysed in serum samples using enzymatic assay on the ADIVA 2400 at KCH.

### Statistical analysis

The data were analysed using SPSS version 20.0 (SPSS Inc, Chicago, IL, USA). For all tests, the significance level was set at *P* < 0.05 (two tailed). All data were normally distributed; they are expressed as mean ± SD or with 95 % CI. Incremental area under the curves (iAUC) was calculated with GraphPad Prism (version 5.01; GraphPad, La Jolla, CA 9203, USA) using the trapezoid rule. Statistical analysis of the data was carried out with repeated-measures analysis of variance, with meal and time as within-subject factors. Post hoc analyses were made using a Bonferroni correction.

## Results

A total of 12 participants completed the study, their details are shown in Table 2. The flow of participants through the study is shown in online supplementary material (Supplementary Figure 1).

**Table 2:** Characteristics of the 12 male subjects

	Mean values ± SD
Age (year)	20.5 ± 1.1
BMI (kg/m^2^)	22.4 ± 2.8
Serum total cholesterol (mmol/L)^a^	3.7 ± 0.4
Serum HDL cholesterol (mmol/L)^a^	1.3 ± 0.3
Serum LDL cholesterol (mmol/L)^a^	2.0 ± 0.4
Serum triacylglycerol (mmol/L)^a^	0.9 ± 0.4
Glucose (mmol/L)^a^	5.1 ± 0.4

^a^Fasting blood samples

**Fig. 1:**
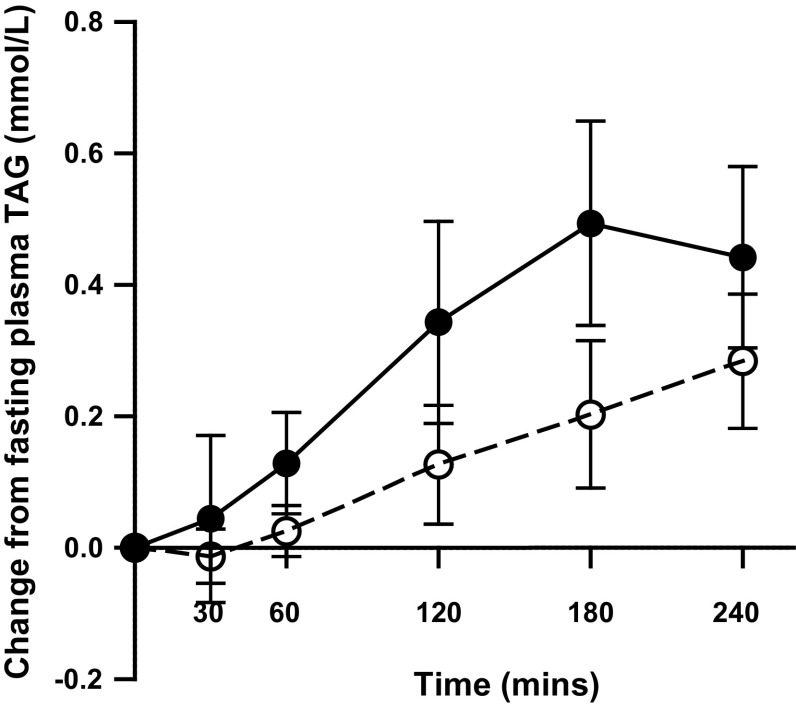
Mean change in plasma triacylglycerol (TAG) and 95 % CIs in healthy men (*n* = 12) after test meals containing 50 g experimental fat from non-IE PSt/PK (*open* *circles*) and IE PSt/PK (*filled* *circles*). Deviations from fasting values were analysed by ANOVA, with the two meals and time (0–240 min) as factors: meal × time interaction *P* = 0.002; meal effect *P* = 0.009

Postprandial serum TAG concentrations (Fig. 1) were significantly lower following non-IE compared with IE (meal effect *P* = 0.009 and meal × time interaction *P* = 0.002) fat blends. Plasma TAG concentration peaked at 3 h following the IE fat and continued to increase at 4 h following the non-IE fat, whereas plasma TAG concentrations started to decline at 4 h following the IE fat. Following non-IE, plasma concentrations were 0.43 mmol/L (95 % CI of the mean difference 0.21, 0.65) lower at 3 h compared with IE (*P* = 0.03). The iAUC for plasma TAG was 51 % (95 % CI 28, 75) lower (*P* = 0.001) following the non-IE blend [31 mmol/L min (95 % CI 16, 46)] versus the IE blend [72 mmol/L min (95 % CI 50, 94)]. Plasma concentrations of the major fatty acid constituents of the test meals (palmitic and oleic acid) did not differ between meals (data not shown). Plasma palmitic acid followed a similar pattern of response to plasma TAG reaching peak concentrations at 3 h following the IE fat (of 1.4 mmol/L; 95 % CI 1.0, 1.8) and 4 h following the non-IE fat (of 1.2 mmol/L; 95 % CI 1.0, 1.45), but the iAUC was not significantly different between fats.

**Fig. 2:**
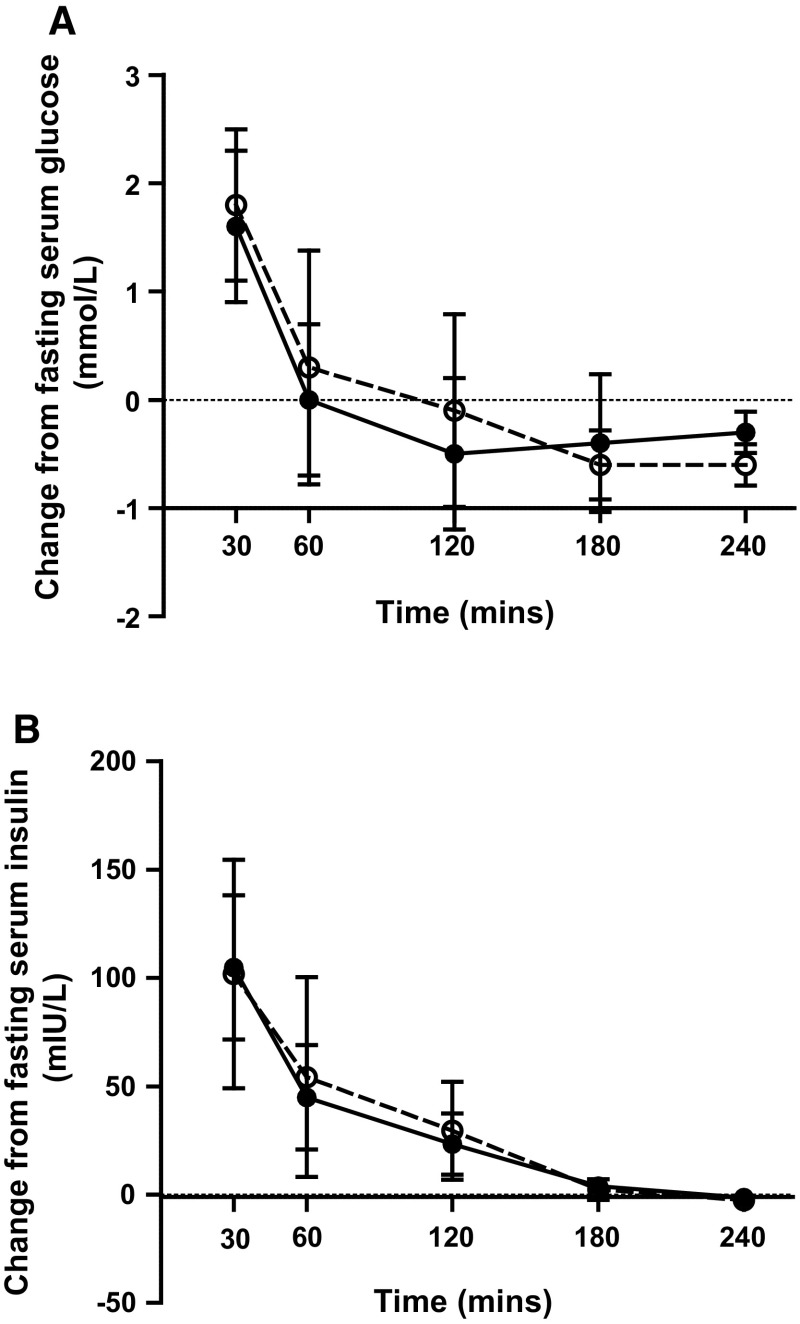
Mean change in serum **a** glucose and **b** insulin concentrations and 95 % CIs in healthy men (*n* = 12) after test meals containing 50 g experimental fat from non-IE PSt/PK (*open* *circles*) and IE PSt/PK (*filled* *circles*). Deviations from fasting values were analysed by ANOVA, with the two meals and time (0–240 min) as factors: time effects for both *P* < 0.001

There were no significant differences in the pattern of the glucose or insulin responses (Fig. 2a, b) between test fats and no differences in the iAUC’s. Serum glucose concentrations increased significantly following the non-IE and IE fat above fasting levels (5.44 and 5.47 mmol/L, respectively) at 30 min (7.26 and 7.06 mmol/L, respectively) and returned to baseline values at 60 min (time effect *P* < 0.001). Insulin concentrations increased significantly following the non-IE and IE fat above fasting levels (8.4 and 10.1 mIU/L, respectively) reaching peak concentrations at 30 min (110.0 and 115.0 mmol/L, respectively); time effect *P* < 0.001. PYY and GIP concentrations (Table 3) increased postprandially following both test meals (time effect *P* < 0.001 for both), but there were no significant differences between test meals. Paracetamol concentrations (Table 3) increased significantly following both test meals (time effect *P* < 0001) but did not differ between meals; iAUC non-IE 1678 mg/L min (95 % CI 1517, 1838) and IE 1656 mg/L min (95 % CI 1470, 1843).

**Table 3:** Plasma PYY (ng/L), GIP (ng/L) and paracetamol (mg/L) concentrations following consumption of interesterified and non-interesterified fat blends (0–240 min)

Time (min)^a^	Non-IE PSt/K	IE PSt/PK
GIP		
0	51 (20, 83)	35 (26, 45)
30	265 (212, 318)	241 (186, 296)
60	283 (212, 354)	260 (207, 134)
120	299 (246, 352)	309 (243, 375)
180	281 (208, 354)	341 (245, 384)
240	201 (160, 242)	208 (155, 261)
PYY		
0	107 (92, 121)	104 (81, 127)
30	122 (110, 134)	134 (110, 159)
60	135 (123, 148)	139 (113, 165)
120	130 (114, 146)	124 (105, 142)
180	124 (112, 136)	124 (110, 138)
240	132 (121, 144)	121 (112, 131)
Paracetamol		
0	0.0 (0.0, 0.0)	0.0 (0.0, 0.0)
30	3.8 (3.1, 4.6)	4.3 (3.6, 5.1)
60	7.0 (5.9, 8.1)	6.8 (5.6, 7.9)
120	9.8 (8.7, 10.8)	9.7 (8.4, 11.0)
180	8.1 (7.4, 8.8)	7.8 (6.6, 8.9)
240	5.9 (5.1, 6.7)	5.9 (5.0, 6.9)

Values are means with 95 % CI

^a^Deviations from fasting values were analysed by ANOVA, with meal type and time as within-subject factors. Time effect for all measures *P* < 0.001

**Fig. 3:**
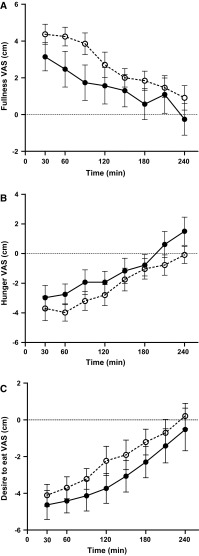
Mean change in VAS satiety scores (cm) with SEM, *n* = 12 after test meals containing 50 g experimental fat from non-IE PSt/PK (*open* *circles*) and IE PSt/PK (*filled* *circles*). Deviations from fasting values were analysed by ANOVA, with the two meals and time (0–240 min) as factors. **a** Fullness; time effect *P* < 0.001; diet effect *P* = 0.034. **b** Hunger; time effect *P* < 0.001. **c** Desire to eat; time effect *P* < 0.001

VAS satiety measurements showed a significant time effect for all (*P* < 0.001 for all), Fig. 3a–c. There was a significant diet effect (*P* = 0.034) for change from baseline in Fullness (‘How full do you feel’), with a higher fullness after the non-IE meal. There were no differences between meals for Hunger (‘How hungry do you feel’) or Desire to eat (‘How strong is your desire to eat’). The total energy intake and weight of food consumed after the ad libitum meals were similar following both test fats; IE 877 kcal (95 % CI 646, 1109); 546 g (95 % CI 402, 690) and non-IE 1028 kcal (95 % CI 726, 1330), 636 g (95 % CI 456, 816).

## Discussion

The primary aim of this study was to investigate the acute effects of one of the most commonly used IE palmitic acid-rich fat blends versus its non-IE counterpart on postprandial lipaemia in order to investigate whether interesterification of the fats that are commercially available might influence cardiovascular risk. Contrary to our hypothesis, we observed a significantly higher postprandial lipaemia following the IE palmitic acid-rich fat. Previous work by our group [11–13] and others [14] has consistently reported a significantly lower (or non-significant lower) postprandial lipaemia following IE palm oil/palm olein versus the non-IE counterpart or a control oil. A possible explanation arises upon examination of the solid fat content and the *sn*-positional composition of the test fats which change following the process of interesterification. In previous studies, single fat sources (palm oil/olein) were interesterified resulting in a higher proportion of palmitic acid in the *sn*-2 position and a higher solid fat content. For example, non-IE palm olein and IE palm olein have a solid fat content at 37 °C of 4 and 15 %, respectively, and % of palmitic acid in the *sn*-2 position of 7 and 37 %, respectively [13]. However, in the current study, a blend of two fats was interesterified; palm stearin (PSt) and palm kernel (PK) (80:20). The non-IE PSt/PK and the IE PSt/PK had a solid fat content at 35 °C of 24 and 21 %, respectively, and % of palmitic acid in the *sn*-2 position of 36 and 54 %, respectively. Thus, interesterification of both palm olein (not commonly consumed) and PSt/PK (widely consumed) fats increased the proportion of palmitic acid in the *sn*-2 position, but only interesterification of palm olein increased the solid fat content at 35–37 °C. In fact, interesterification of the PSt/PK blend slightly reduced the solid fat content at 35 °C. These differences in solid fat are likely to arise from differences in the proportion of tri-palmitin (PPP); for example, non-IE PSt contains approximately 28 % of its TAG species as PPP, which decreases upon interesterification [21], whilst the interesterification of palm olein increases the proportion of PPP from 1 to 8 % [12]. This would also explain the lack of effect of *sn*-positional composition on postprandial lipaemia observed by Summers et al. [22] and Zampelas et al. [23], when test fats containing single low melting point TAG species are fed; 1, 3-oleyl, 2-palmityl (OPO) versus 1, 2-palmityl, 3-oleyl (POO). Our results therefore add to the growing body of evidence that the proportion of solid fat content at 37 °C is a major determinant of the postprandial response, rather than the *sn*-positional composition [1].

It was hypothesised that a high solid fat content may attenuate the postprandial response via effects on rates of gastric emptying which may subsequently influence glycaemic, incretin and satiety responses to the meal. Previously, gastric emptying measured using scintigraphy has been shown to be delayed following consumption of solid fats (hydrogenated vegetable oil; melting point 43 °C) compared to liquid oils (vegetable oil) in healthy volunteers, with the time to emptying 90 % fat/oil following the liquid oil and solid fat at 206 and 264 min, respectively [24]. It is plausible that the solid fat content of the test fats may influence the lipaemic response by effects on coalescence, phase separation and micelle formation and/or access of digestive enzymes to the fat droplets (due to differences in particle size) thus delaying gastric emptying and attenuating the postprandial lipaemic response. However, contrary to our hypothesis, there were no differences in estimated rates of gastric emptying indirectly assessed by paracetamol absorption, despite large differences in lipaemia. This would provide a likely explanation for the lack of difference observed in the glycaemic and gut hormone response, which indicate similar rates of delivery of glucose and fat to the intestine, and similar levels of gastric distention and incretin release. These results concur with current evidence that interesterification of palmitic acid-rich fats does not adversely affect acute changes in glucose or insulin responses [13–15, 23].

Despite the lack of differences in incretin responses and gastric emptying, the VAS scores showed that the non-IE fat resulted in a significantly greater feeling of fullness compared to the IE fat. It is plausible that the higher solid fat content of the non-IE fat may have increased gastric distention due to the retention of the higher melting point TAG species which would stimulate a greater feeling of fullness [25], but this is not supported by the gastric emptying data, or the other VAS scores and requires further investigation.

In the absence of differences in gastric emptying between the test meals, it is possible that the differences in lipaemia were influenced by differences in enzymatic hydrolysis and removal of TAG and remnant particles from the circulation, which are also known to influence the rate and extent of lipaemia, independent of effects on gastric emptying. It had previously been reported in animal and infant studies that SFA in the *sn*-2 position enhances absorption [26] and delays hydrolysis of TAG by lipoprotein lipase (LPL) [27], suggesting that IE palmitic acid-rich fats (with an increased proportion of *sn*-2 palmitic acid) may be absorbed more rapidly and cleared more slowly from the circulation and enhance the postprandial response. However, Summers reported no arterio-venous differences across adipose tissue following fats with palmitic acid predominantly in the *sn*-2 position versus the *sn*-1 and -3 positions [22], suggesting that there is no effect of *sn*-positional composition on LPL activity and release of fatty acids into venous plasma or storage in adipocytes. Indeed, we have previously reported no difference in post-heparin lipoprotein and hepatic lipase activity following palmitic acid-rich fats versus a control oil, with different solid fat contents, despite differences in postprandial lipaemic response [13]. Whilst it would be of interest to measure rates of hydrolysis by lipoprotein lipase and pancreatic lipase (in vitro using simulated gastric models), it was beyond the scope of this study.

The current trial was planned as an exploratory investigation into postprandial lipaemic responses to these never-before studied commercially available fats. The study design has limitations, including the method used to measure gastric emptying. Measurement of the absorption of paracetamol following a 1.5-g oral load was used in the current study as it is a relatively simple and inexpensive method and has been shown to correlate with the ‘gold standard technique’ of scintigraphy [28]. However, paracetamol is water-soluble and therefore empties with aqueous components in the meal, and consequently any differences in the amount of solid fat retained in the stomach between meals might not be revealed using this method [29]. Another key limitation is that postprandial measurements were only made up to 4 h. It is possible that although the IE fat caused a rapid increase in lipaemia (peaking at 3 h), it might have returned to fasting values at a different rate to the non-IE fat (as suggested by the decline it TAG concentrations at 4 h following the IE fat). Although there is robust epidemiological evidence for the relationship between elevated non-fasting TAG and risk of CVD [16–18], it remains uncertain as to whether the duration of lipaemia (time to return to baseline) or the peak TAG (Cmax) response has a greater impact on subsequent risk of CVD [via effects on postprandial inflammation, oxidative stress, impaired vascular function and lipoprotein remodelling (small dense LDL, reduction in HDL concentration and cholesterol rich remnant particles)]. Despite its limited duration, the current study shows a 0.43-mmol/L difference in 3 h plasma TAG concentrations which is considered a clinically relevant difference; previous prospective cohort studies have observed significant differences in hazard ratios (multivariable adjusted) for CVD mortality [18], CVD events [16] and IHD [17] between quintiles of non-fasting TAG (differences in quintiles of approximately 0.3 mmol/L) in the postprandial range of TAG observed in this study. Therefore, the difference in postprandial TAG concentrations following IE PSt/PK requires further exploration over a longer postprandial period to determine associations with acute cardio-metabolic risk markers and to elucidate the underlying mechanisms causing the differences in lipaemic response between IE and non-IE fats. It is also worth taking into consideration that the fatty acid composition of the background diet can influence the postprandial lipaemic response to standard test meals [30, 31]. Therefore, outcomes of single test meal studies of dietary fatty acids may not necessarily be extrapolated to effects of chronic intake since lack of equilibration between tissue fatty acid pools reduces the chance of detecting effects of specific fatty acids and specific structural changes on postprandial CVD risk markers [32]. Lastly, it is also noteworthy that this study was conducted on young healthy males. It is well known that different populations exhibit differences in postprandial lipaemic responses; for example sex, obesity, age, fasting TAG and insulin sensitivity influence the postprandial response to a standard test meal [19]. Further research is therefore needed to evaluate the acute effects of IE fats in different population groups that are susceptible to elevated or prolonged postprandial lipaemic responses.

In conclusion, results from the current study show that a commercially relevant IE palmitic acid-rich fat used in many food applications throughout Europe results in a significantly higher early phase postprandial lipaemic response compared to its non-IE counterpart. Since elevated non-fasting TAG is associated with an increased risk of CVD, and these IE fat blends are estimated to supply between 2 and 10 % of average daily energy intake, there is a pressing need to characterise their metabolic effects and investigate whether there is any impact on long-term cardio-metabolic health.

## Electronic supplementary material

Below is the link to the electronic supplementary material.

Supplementary material 1 (DOCX 29 kb)
