# Supplementary material for: Modulation of postprandial lipaemia by a single meal containing a commonly consumed interesterified palmitic acid-rich fat blend compared to a non-interesterified equivalent
Source: Eur J Nutr. 2016 Aug 10;56(8):2487–95. doi: 10.1007/s00394-016-1284-z (PMC5682848; doi:10.1007/s00394-016-1284-z)
Supplement: Supplementary file 1 — Supplementary material 1 (DOCX 29 kb) [file 394_2016_1284_MOESM1_ESM.docx]

Withdrew

n=4

- n=1 time constraints
- n=1 unable to fully consume the test meal
- n=1= vasovagal syncope
- n=1 personal reasons

Screened n=17

Eligible n=16

**Study day 1**

**Study day 2**

Data available for analysis n=12

Declined to participate n=1

Randomly assigned to treatment n=16
